# Retarded *Onchocerca volvulus *L1 to L3 larval development in the *Simulium damnosum *vector after anti-wolbachial treatment of the human host

**DOI:** 10.1186/1756-3305-5-12

**Published:** 2012-01-11

**Authors:** Anna Albers, Mathias Eyong Esum, Nicholas Tendongfor, Peter Enyong, Ute Klarmann, Samuel Wanji, Achim Hoerauf, Kenneth Pfarr

**Affiliations:** 1 Institute for Medical Microbiology, Immunology and Parasitology, University Hospital Bonn, Sigmund-Freud-Str. 25, D-53105 Bonn, Germany; 2 Research Foundation for Tropical Diseases and the Environment, P.O. Box 474, Buea, Cameroon; 3 Department of Plant and Animal Sciences, Faculty of Science, University of Buea, P.O. Box 63, Buea, Cameroon; 4 Department of Biochemistry and Microbiology, Faculty of Science, University of Buea, P.O. Box 63, Buea, Cameroon; 5 Institute for Medical Biometry, Informatics and Epidemiology, University Hospital Bonn, Sigmund-Freud-Str. 25, D-53105 Bonn, Germany

**Keywords:** *Onchocerca volvulus*, *Wolbachia*, doxycycline, development, onchocerciasis

## Abstract

**Background:**

The human parasite *Onchocerca volvulus *harbours *Wolbachia *endosymbionts essential for worm embryogenesis, larval development and adult survival. In this study, the development of *Wolbachia*-depleted microfilariae (first stage larvae) to infective third stage larvae (L3) in the insect vector *Simulium damnosum *was analysed.

**Methods:**

Infected volunteers in Cameroon were randomly and blindly allocated into doxycycline (200 mg/day for 6 weeks) or placebo treatment groups. After treatment, blackflies were allowed to take a blood meal on the volunteers, captured and dissected for larval counting and DNA extraction for quantitative real-time PCR analysis.

**Results:**

PCR results showed a clear reduction in *Wolbachia *DNA after doxycycline treatment in microfilariae from human skin biopsies with > 50% reduction at one month post-treatment, eventually reaching a reduction of > 80%. Larval stages recovered from the insect vector had similar levels of reduction of endosymbiotic bacteria. Larval recoveries were analysed longitudinally after treatment to follow the kinetics of larval development. Beginning at three months post-treatment, significantly fewer L3 were seen in the blackflies that had fed on doxycycline treated volunteers. Concomitant with this, the proportion of second stage larvae (L2) was significantly increased in this group.

**Conclusions:**

Doxycycline treatment and the resulting decline of *Wolbachia *endobacteria from the microfilaria resulted in retarded development of larvae in the insect vector. Thus, anti-wolbachial treatment could have an additive effect for interrupting transmission by reducing the number of L3 that can be transmitted by blackflies.

## Background

Onchocerciasis, caused by *Onchocerca volvulus*, is endemic in many sub-Saharan countries with further foci in Latin America and Yemen [1,2]. The number of infections is estimated to be ~ 37 million [3,4]. The chronic nature and morbidity of onchocerciasis is associated with microfilariae (first stage larvae; Mf) that migrate through the skin and the eye. When the Mf die, the immune response to the dead larvae can result in dermatitis, skin atrophy and inflammation in the eyes. The latter can progress into reduced vision and blindness. Vector control and mass treatment with ivermectin, a strong microfilaricidal drug which can produce temporary sterility [5], have been successfully used to regionally reduce the burden of parasite infection. Due to the resumption of fertility after interruption of ivermectin treatment, the drug has to be administered for many years. Studies in Ghana have identified *O. volvulus *populations that are less responsive to ivermectin [6-10], therefore identification of new drug regimens is required before ivermectin resistance may develop and spread. Ideally a new drug would have macrofilaricidal and/or permanent sterilising activity.

In recent years, key drug trials have been performed with a new chemotherapeutical approach to anti-filarial therapy, the targeting of the essential *Wolbachia *endobacteria present in many filariae with the antibiotic doxycycline. This approach has resulted in long-term sterilisation of adult female worms in onchocerciasis [11-14]. More importantly, anti-wolbachial therapy also results in a macrofilaricidal effect in *O. volvulus *[13,15,16].

Doxycycline affects several stages in the parasite life cycle. Embryonic stages from morulae to coiled Mf are the most sensitive to *Wolbachia *depletion [13,15,16]. The development from L3 (infective larval stage) into adult worms is also affected [17-20]. In the mammalian host, Mf are apparently unaffected by the decreased endobacterial load [12,17]. However, the effect of *Wolbachia*-depletion on *O. volvulus *larval development from Mf to L3 in the obligate arthropod vector remains unknown. Arumugam et al. showed that larvae of the rodent filaria *Litomosoides sigmodontis *were dependent upon the endosymbionts for development into infective L3 larvae in the mite vector [17]. Fewer female worms were able to develop from *Wolbachia *depleted Mf, because of their need of a higher threshold level of endobacteria to survive.

This study was designed to investigate whether *Wolbachia *endobacteria are essential for the transmission of onchocerciasis. To determine this, we analysed the role of *Wolbachia *in the development of *O. volvulus *Mf into L3 infective larvae in the *Simulium *vector by depleting the endobacteria from the Mf prior to their ingestion by blackflies during a blood meal.

## Methods

### Ethical approval

This study received ethical clearance from the Institutional Review Board of the Tropical Medicine Research Station, Kumba and was conducted in accordance with the Helsinki Declaration of 1975 as revised in 1983, 2000 and 2002.

### Selection of study patients and treatment

**Figure 1:**
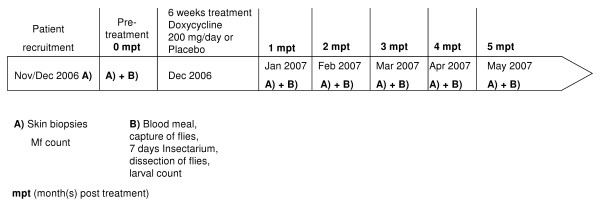
**Study timeline of recruitment, treatment and analysis**. Patients were recruited in November/December 2006. After the informed consent was signed by each volunteer, skin biopsies were taken from which Mf were isolated and counted. Volunteers were allocated into two treatment groups. Before treatment with doxycycline or placebo (pre-treatment, month 0), volunteers were brought to the capture side to allow *Simulium damnosum *flies to take a blood meal. Flies were captured and raised in an insectarium for seven days. After dissecting the flies *Onchocerca volvulus *larvae were counted. This was repeated monthly for five months after doxycycline treatment was completed.

In November and December 2006, participants for the study were recruited in four neighbouring villages in an onchocerciasis-endemic region of South West Cameroon (Figure 1). Patients for the study were selected from male volunteers, aged 21 to 50 years, after informed consent was signed. Exclusion criteria were abnormal hepatic (SGPT and SGOT) and renal (creatinine) functions, persons with chronic infections and under prolonged medication, alcohol abuse and intolerance to doxycycline. As there was mass treatment with ivermectin in the area in April 2006, all patients were asked whether they took their doses at this time or any time before, but all volunteers answered in the negative. Inclusion criteria were absence of other clinically manifested diseases as assessed by a medical doctor and ≥ 10 Mf in skin biopsies. Two bloodless skin biopsies, one from each iliac crest of each participant, were aseptically obtained using a Holth corneoscleral punch. Each biopsy was immersed in 2 drops of 0.9% NaCl solution in a separate well of a labelled 96-well round-bottom microtiter plate. The plates were transported to the laboratory and skin biopsies were incubated overnight at room temperature to allow the emergence of Mf into the saline solution. The Mf were counted using 10-fold magnification of a microscope and the number of Mf were expressed as Mf per skin biopsy.

Twelve volunteers were blindly allocated into two treatment groups. One group of 7 volunteers received doxycycline (200 mg/day) for 6 weeks while the second group of 5 volunteers received matching placebo.

### Infection of wild *Simulium *flies with *Onchocerca volvulus*

Before and after treatment, the volunteers were brought to a pre-selected *O. volvulus-*free capture point near a *Simulium *breeding site on the bank of the River Mungo to allow *Simulium damnosum *flies, the vector of *O. volvulus*, to take a blood meal. The site was chosen based on the results of dissection of wild *Simulium *flies to determine the natural parous, infection and infective rates, the latter two variables indicating that there was no *O. volvulus *endemic to the capture site (Table 1). These parameters were monitored throughout the study period. The fed flies were captured on the volunteers in small plastic containers. Containers for each volunteer were kept separately in plastic baskets. At least four hundred flies were caught from each patient in four days at each time point. Transport to the insectarium was carried out in cold boxes. The flies were then kept for 7 days at 25°C and 80% relative humidity to allow the Mf to develop into infective third stage larvae (L3).

**Table 1:** Endemicity of *Onchocerca volvulus *infection in *Simulium damnosum *flies, Mungo river Etam, Cameroon

Date	Flies captured	Flies dissected	Parous (%)	Nulliparous (%)	Infected (%)	Infective (%)
June 2006	702	702	256 (36.5)	446 (63.5)	2 (0.78)	0 (0.0)

July 2006	483	483	46 (9.5)	437 (90.5)	0 (0.0)	0 (0.0)

September 2006	537	537	36 (6.7)	501 (93.3)	0 (0.0)	0 (0.0)

Total	1722	1722	338 (19.6)	1384 (80.4)	2 (0.59)	0 (0.0)

Larvae were isolated from the flies by dissection using a dissecting microscope and their developmental stages were scored. The number of flies dissected at each time point varied due to different fly mortality rates. The isolated developmental stages of the larvae (L1, L2 and L3) were stored separately in 80% ethanol. At least two tubes each containing different numbers of L1, L2 and L3 isolated from flies fed on each volunteer at each time point were stored for later DNA extraction and quantification of *Wolbachia*.

### Follow-up of volunteers and monitoring of Mf load after treatment

During the following 5 months post-treatment, flies were captured on a monthly basis on the volunteers as described above (Figure 1). Skin biopsies were also obtained from the patients at each time point to assess their Mf load after the antibiotic treatment. After counting, the Mf were also stored in 80% ethanol for later quantitative PCR analysis to determine their *Wolbachia *content.

### Kinetics of larval development after anti-wolbachial therapy

The total number of L1 (first larval stage), L2 (second larval stage) and L3 (third, infective larval stage) collected after dissection of the flies was calculated for doxycycline and placebo treated individuals. The proportion of each larval stage generated for that month in each group was calculated and plotted against the month of dissection to follow the kinetics of the development of larvae in both treatment groups. At pre-treatment and one month follow-up, the L1 and L2 larvae counts were combined.

### DNA extraction from larvae

One tube each of the different stages of larvae (L1, L2 and L3) per time point preserved in ethanol was randomly selected for DNA extraction and quantification of *Wolbachia*. The larvae preserved in ethanol were first centrifuged at 1800 rpm (Multifuge 4KR, LH4000-75006475, Heraeus, Haunau, Germany) for five minutes. The ethanol was slowly removed with a pipette without disturbing the larvae, leaving 100 μl of volume. These larvae were then suspended in the remaining ethanol and transferred to a new tube.

DNA was extracted using the QIAamp^® ^DNA mini Kit (Qiagen, Hilden, Germany) following the tissue protocol of the kit with the following modifications to achieve the maximum amount of quality DNA. The incubation period with proteinase K (Qiagen) was extended from 10 min to overnight at 56°C. Wash buffers 1 and 2 were increased to 700 μl followed by elution with 2×50 μl of AE buffer. For each elution step, the columns were incubated with AE buffer for 5 minutes at room temperature prior to centrifugation.

### Quantitative real-time PCR

To determine the amount of *Wolbachia *in the larvae isolated from the vector captured after having fed on doxycycline or placebo treated individuals, the purified DNA samples of randomly selected larvae for each volunteer and time point were analysed by quantitative real-time PCR. Primers and hybridisation probe were designed with Primer3 software [21] for the *O. volvulus Wolbachia ftsZ *gene (GenBank accession No. AJ276501), which codes for a single-copy cell division protein. For all samples, the following master mix was used: 1 × QuantiTect^® ^Virus NR Master Mix (Qiagen), 300 nM each forward and reverse primers (Table 2), 50 nM TaqMan hybridisation probe with the fluorescent dye 6-FAM (6-carboxyfluorescein) and Tamra (Biomers, Ulm, Germany) and 10 μl of sample DNA in a 20 μl reaction. The PCR program utilised a two-step reaction of the following conditions in a Rotor Gene 6000 (Corbett Research, Sydney, Australia): 1 × 5 min at 95°C, 45 cycles of 94°C for 10s and 58°C for 45s. Fluorescence was acquired on the FAM channel at 58°C. A second PCR was performed to quantify *O. volvulus β-actin *(GenBank accession no. M84916) to normalise the *ftsZ *values [22]. The optimised PCR conditions were: 1 × HotStar^® ^Taq Polymerase buffer (Qiagen), 4 mM MgCl_2_, 200 μM dNTP, 300 nM each forward and reverse primers (Table 2), 0.2 μl of Sybr Green (1:1,000 diluted in DMSO, Roche, Mannheim, Germany), 2.5 U HotStar^® ^Taq Polymerase and 2 μl DNA in a 20 μl reaction. The PCR profile used a 3-step program with an initial 95°C for 15 min, followed by 35 cycles at 94°C for 10s, 52°C for 15s and finally 72°C for 15s. Fluorescence was acquired on the FAM channel at the end of the extension step.

**Table 2:** Primer and Probe sequences for quantification of the *Wolbachia ftsZ *and *Onchocerca volvulus β-actin *genes

Gene target	Name	Sequence 5'-3'
*O. volvulus Wol ftsZ*	Forward	aggaatgggtggtggtactg
*O. volvulus Wol ftsZ*	Reverse	ctttaaccgcagctcttgct
*O. volvulus Wol ftsZ*	Hybridization probe	ccttgccgctttcgcaatcac
*O. volvulus β-actin*	Forward	gtgctacgttgctttggact
*O. volvulus β-actin*	Reverse	gtaatcacttggccatcagg
Murine Interferon γ	Forward	tcaagtggcatagatgtggaagaa
Murine Interferon γ	Reverse	tggctctgcaggattttcatg

In both PCR assays, each sample was analysed in triplicate. Every run contained a plasmid containing sequences specific for *Wolbachia ftsZ *or *O. volvulus actin *genes with a known number of copies for use as a standard. A no-template control of water instead of sample DNA was used as negative control. Copy numbers for each gene were calculated using a modification of the comparative quantification formula as described previously [23]. Using the Rotor Gene 6000 version 6.0 software the amplification (A) of each sample was calculated and the mean taken from replicate samples (sam). A plasmid containing either the *ftsZ *or *β*-*actin *sequence was quantified and copies/μl determined for use as a reference (ref) in each PCR run. The amplification factor of the sample compared to the specific reference was then calculated using the following formula: A_ref_^take off_ref_/A_sam_^take off_sam_. The take off is defined as the crossing point cycle where all measured samples are 20% above background. The amplification factor was then multiplied by the known concentration of the reference to give copies/μl. The *ftsZ*/*β-actin *ratio was then calculated.

To exclude inhibition of the PCR reaction by inhibitors in the DNA a third PCR with a plasmid containing a fragment of the murine Interferon-γ gene was performed in the presence of the extracted DNA. The samples were set up for real-time PCR with a master mix containing 1 × HotStar^® ^Taq Polymerase buffer (Qiagen), 200 μm dNTP, 400 nM each of forward and reverse primer (Table 2), 0.2 μl SYBR^® ^Green (1:1000 diluted in DMSO, Roche, Mannheim, Germany), 2.5 units HotStar^® ^Taq Polymerase, 2 μl reference Plasmid DNA and 2 μl sample DNA in a 20 μl reaction. The PCR profile was: 1 × 15 min at 95°C, 45 cycles of 94°C for 15s, 58°C for 20s, 72°C for 20s. Fluorescence was acquired on the FAM channel. An increase in cycle number of ≥ 1 cycle over the plasmid-only sample indicated the presence of PCR inhibitors in the DNA extracted from the larvae.

### Data analysis and statistics used

To test the interaction of treatment and time, we calculated the difference between follow-up time-points and pre-treatment regarding the percentages of L3-larvae to account for the development between pre-treatment and after treatment in each patient. A repeated measures ANOVA was then performed using PASW 18 (IBM, Chicago, USA). The proportions of different larval stages were analysed by calculating the L3/total larvae and L1+L2/total larvae ratios. Statistical significance between larvae from vectors fed on doxycycline or placebo treated individuals was determined with the Mann Whitney U test (P ≤ 0.05 was considered significant) using PASW 18. Results were presented using the median. Larval recoveries were expressed as median with 10-90^th ^percentiles. *Wolbachia *quantities determined by PCR were expressed as the ratio of *ftsZ*/*β-actin*.

## Results

### Selection of *Onchocerca volvulus*-free *Simulium *capture site

Before bringing *O. volvulus *infected volunteers to the blackfly capture site, we determined the natural parous and infection rates by collecting and dissecting wild *Simulium *flies. A total of 1722 female flies were collected in June, July and September of 2006. The flies were dissected and *O. volvulus *infection status determined: 19.6% were parous (females are reproductively mature) of which 2 (0.59%) were infected, but did not contain infective stage larvae (Table 1). Thus, the local blackfly population was sexually mature, seeking blood meals, and their river location was essentially free of endogenous *O. volvulus *infections.

### Kinetics of larval development

**Figure 2:**
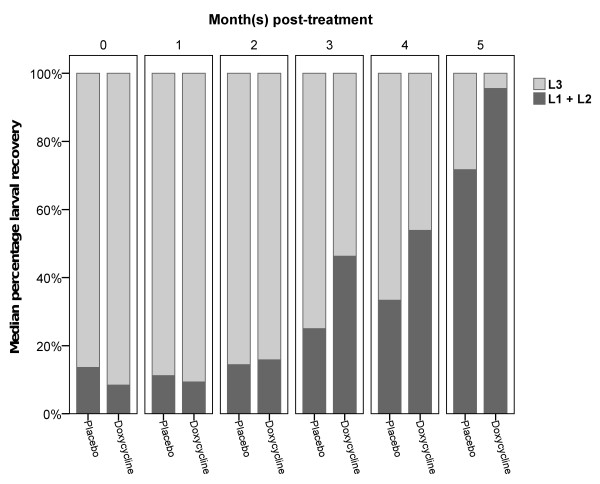
**Depletion of *Wolbachia *from *Onchocerca volvulus *larvae reduces the number of larvae that develop into the infective stage in the *Simulium *vector**. The proportion of larvae at each stage was calculated by dividing the number of larvae (stage L1+L2; L3) by the total larvae collected from the captured blackflies after they had fed on the doxycycline (n = 7) or placebo (n = 5) treated volunteers. The proportion of L1+L2 larvae in flies that had fed on doxycycline treated individuals (black bars) was higher beginning at 3 months post-treatment compared to placebo. The proportion of L3 larvae in flies that had fed on doxycycline treated volunteers (white bars) was lower than that in the placebo volunteers at 3, 4 and 5 months post-treatment. Proportions are given as percentages of median larval recovery. 100% corresponded to the total number of larvae recovered at each indicated time.

To determine the skin microfilarial loads and recovery of *O. volvulus *larvae from flies that had fed on doxycycline or placebo treated individuals, skin biopsies and dissected flies were analysed for developing larvae. Mf were counted and expressed as Mf per skin biopsy. The median number of L1, L2, and L3 larvae collected after fly dissection was calculated per treatment group (Table 3). Because the number of flies that were dissected from each volunteer was different due to different numbers recovered at the biting site and different survival rates in the insectarium, the proportions of each larval stage that developed in each treatment group were plotted against the month of dissection (month post-treatment) to follow the kinetics of larval development (Figure 2). A repeated measures ANOVA determined that there was a significant difference in L3 recovery between the 5 measurement time-points (P < 0.001, Pillai-Spur) but no clear interaction between the measurement time-points and the treatment (P = 0.092, Pillai-Spur), although the doxycycline group was different (P = 0.005).

**Table 3:** Larval recoveries from skin snips and blood-fed black flies of doxycycline and placebo treated volunteers

	Mf		L1/L2		L3	
**Month**^**a**^	**Doxy**^**b**^ **n = 7**	**Placebo** **n = 5**	**Doxy** **n = 7**^**c**^	**Placebo** **n = 5**	**Doxy** **n = 7**	**Placebo** **n = 5**

0	26^d^ (13-80)	25 (10-76)	52 (45-157)	124 (71-155)	703 (509-1214)	789 (396-941)

1	42 (32-148)	83 (31-221)	29 (3-178)	33 (7-69)	281 (150-607)	313 (226-516)

2	40 (8-92)	114 (15-192)	133 (28-524)	112 (42-294)	797 (211-1615)	666 (385-963)

3	13 (7-40)	27 (10-113)	678 (167-965)	390 (90-877)	709 (335-1070)	983 (487-1169)

4	9 (2-127)	63 (9-92)	397 (103-1218)	217 (118-625)	318 (71-593)	406 (247-542)

5	28 (4-83)	63 (4-105)	426 (201-1476)	575 (388-1128)	53 (17-320)	240 (116-504)

^a ^Month after treatment (Group 0 is pre-treatment)

^b ^Treatment with Doxycycline (200 mg/day) for 6 weeks

^c ^At 2 months post treatment only 6/7 doxycycline treated volunteers attended the follow-up meeting

^d ^Median (range)

Since there was no clear interaction, pair-wise comparisons between the treatment groups at each time-point were performed. Starting three months post-treatment, differences in the median recovery of L1+L2 and L3 from flies fed on the doxycycline treated volunteers were seen. The proportions of L1+L2 larvae recovered from flies that had fed on doxycycline treated individuals were higher at 3, 4 and 5 months post- treatment (Figure 2). At three and five months post-treatment, the differences were significant compared to placebo (P < 0.05, Mann-Whitney U test). Correspondingly, beginning at three months post-treatment, a decrease in the proportion of L3 that developed in flies that had fed on doxycycline treated individuals as compared to placebo treated individuals was observed, with a significant difference seen for months 3 and 5 (P < 0.05, Mann-Whitney U test; Figure 2).

### *Wolbachia *depletion after treatment with doxycycline

To analyse the effect of treatment with antibiotic on the bacterial endosymbionts of *O. volvulus *Mf from the skin and larvae isolated from the vector, a quantitative real-time PCR assay was established for the *O. volvulus Wolbachia ftsZ *gene. The detection limit of the PCR was increased to 30 *ftsZ *copies (1 *ftsZ *= 1 *Wolbachia*) compared to our previous study [13]. For normalisation to worm material in the individual sample, a second PCR assay for *O. volvulus β-actin *was performed [22].

Compared to the placebo group, fewer samples were positive for *ftsZ *above the PCR detection limit in the Mf from doxycycline treated individuals at 2, 3, 4 and 5 months post-treatment. Analysis of the Mf from skin biopsies showed a > 50% reduction in the number of *Wolbachia *one month post-treatment in the doxycycline treated group (Table 4). *Wolbachia *reduction further increased to 81% in the follow-up time points. At the two month follow-up only 2/6 Mf samples (33%) gave a positive *ftsZ *signal in the PCR, and further decreased to only 1/7 samples (14%) being *ftsZ *positive at the 5 month follow-up. A similar pattern was seen in all larval stages collected from the vector. In contrast, in the placebo group, almost every larval stage at each time point produced a clear signal above the detection limit for bacterial *ftsZ*. Only two placebo samples (L1 at pre-treatment and L2 at month 2 post-treatment) did not have a signal above the *ftsZ *detection limit. These samples also gave a weak signal for *actin *(data not shown), indicating a very low DNA content. For all samples, a PCR test for inhibition did not show any inhibition by the sample. The negative *ftsZ *samples therefore did not result from inhibitory factors in the DNA.

**Table 4:** Effect of doxycycline and placebo treatment on *Wolbachia *loads in larval worms: results quantitative PCR

	Mf (skin)		L1 (vector)		L2 (vector)		L3 (vector)	
**Month**^**a**^	**Doxy**^**b**^	**Placebo**	**Doxy**	**Placebo**	**Doxy**	**Placebo**	**Doxy**	**Placebo**

0	7/7^c^ (0.06)^d^	5/5 (0.02)	7/7 (0.058)	4/5 (0.03)	^e^	^e^	6/6 (0.026)	5/5 (0.05)

1	6/7 (0.024)	4/4 (0.05)	4/7 (0.002)	5/5 (0.06)	^e^	^e^	3/7 (0.008)	5/5 (0.03)

2	2/6 (0.068)	5/5 (0.22)	0/6	5/5 (0.11)	1/6 (0.007)	4/5 (0.06)	2/6 (0.011)	5/5 (0.05)

3	2/7 (0.085)	4/4 (0.70)	2/7 (0.06)	5/5 (0.41)	4/7 (0.029)	5/5 (0.16)	4/7 (0.059)	5/5 (0.19)

4	3/7 (0.085)	5/5 (0.30)	0/7	4/4 (0.32)	3/7 (0.002)	4/4 (0.16)	2/7 (0.036)	5/5 (0.43)

5	1/7 (0.167)	5/5 (0.56)	3/7 (0.001)	3/3 (0.08)	1/7 (0.002)	4/4 (0.09)	3/6 (0.017)	5/5 (0.12)

^a ^Month after treatment

^b ^Treatment with Doxycycline (200 mg/day) for 6 weeks

^c ^Number of *Wolbachia ftsZ *positive volunteers

^d ^Median *ftsZ/β-actin *ratio

^e ^L1 and L2 counts were combined at months 0 and 1 of the study

## Discussion

In previous studies much work has been done showing the requirement of *Wolbachia *for oogenesis, embryogenesis and adult worm survival in infections with the filarial parasite *O. volvulus *[11,12]. Other studies have analysed the effects of tetracycline on larval development in the mammalian host. Tetracycline administered concomitantly with the start of infection with *L. sigmodontis *led to significant growth retardation in worms [24]. Oral treatment with tetracycline inhibited *Brugia pahangi *and *Brugia malayi *development from L3 to adult worms in Mongolian gerbils [18-20,25].

Different to animal models, little is known in human infection about the effects of antibiotic treatment of the mammalian host on the larval development in the insect vector. Therefore, the focus of this study was to analyse whether Mf from *O. volvulus *infected patients who had been treated with doxycycline, and thus had reduced or absent *Wolbachia *levels, could still develop into the infective third stage larvae (L3) in the *Simulium *vector.

*Simulium *flies were allowed to bite *O. volvulus *infected volunteers, then captured, raised in an insectarium and then dissected to count larvae that had developed. Prior to bringing the infected volunteers to the capture site, wild *Simulium *flies were captured and dissected to determine the natural parous, infection and infective rates. The very low infection (0.59%) of the wild *Simulium *flies minimised the chance of an already infected fly feeding on the study volunteers (Table 1).

Our results show that Mf treated with doxycycline, and therefore depleted of their *Wolbachia*, developed into L3 at a considerably lower rate than Mf from placebo treated patients during the observation period. This was most apparent as a decrease of L3 with a corresponding increase of L1 and L2 seen in the doxycycline group at 3, 4 and 5 months post-treatment (Figure 2). Whether the few remaining L3 with low numbers of *Wolbachia *might be able to establish an infection in humans remains an open question that is technically and ethically impossible to answer for *O. volvulus *infections.

To support the requirement of *Wolbachia *during larval development, the depletion of the endobacteria was verified by a quantitative PCR assay for the *Wolbachia ftsZ *gene. The PCR results showed a clear reduction of *Wolbachia ftsZ *in the Mf after one month of antibiotic treatment (52% compared to placebo), with Mf from fewer than 50% of volunteer samples even having detectable *ftsZ *at 2, 3, 4 and 5 months post-treatment (Table 4). In the few doxycycline treated larval samples that had an *ftsZ *signal, there was a clear reduction of the *ftsZ/β-actin *ratio. Because so many of the treated samples were negative for *ftsZ*, the sample size was too small for a statistical test.

In addition to the above described effect on larval development in the insect vector, depletion of the *Wolbachia *from *O. volvulus *Mf could have two other beneficial effects. One possible benefit is a reduced chance of *Wolbachia*-depleted larvae establishing an infection. This is supported by work with the *L. sigmodontis *murine model of filarial infections and results from the fact that *Wolbachia *increase the number and degranulation of mast cells at the site of infection, resulting in greater vascular permeability [26]. This effect is linked to the TLR-2 innate immune receptor, which recognizes and is activated by *Wolbachia *molecules [27-29]. The second possible benefit is the reduction in pathology in already infected individuals. Mf are the primary inducers of pathology due to their induction of a strong immune response when they die [30]. Work with animal models and natural animal *Onchocerca *infections have shown the importance of *Wolbachia *in the nematodes for attracting immune cells to the site of infection and more importantly, a reduction in the damage done to the eye when the endobacteria are depleted [30-33].

Changes in the number of L3 that developed from *Wolbachia*-depleted Mf did not appear until 3 months post treatment. We hypothesize that whatever products that the endobacteria provided to the nematodes for proper development are stable for two months and had already been produced and made available to the Mf prior to the depletion of the *Wolbachia*. When these Mf are taken up by the vector, they are then able to continue their development until the endobacterial substances are depleted. A better understanding of this phenomenon will be developed as the basis for the requirement of *Wolbachia *by filarial nematodes is elucidated.

Although not significant, within the placebo group the number of L3 larvae recovered from the *Simulium *decreased over time post-treatment. This could be due to the fall in temperatures (from 28°C to 24°C) in the area at these time points reducing the capacity of the *Simuliae *to support the infective L3 [34,35]. However, this has to be confirmed in other experiments. The results of a repeated measures ANOVA allowed us to conclude that there was no interaction between the time of measurement and treatment. Nevertheless, there is a significant difference in the L3 reduction observed between the doxycycline and the placebo treated volunteers (Figure 2).

Inhibition of larval development after antibiotic treatment has been observed in several other studies. In a murine model with *L. sigmodontis*, significantly fewer L3 developed in the intermediate host if Mf were depleted of their *Wolbachia *endobacteria with tetracycline treatment [17]. Furthermore, infection of jirds with *Wolbachia*-depleted L3 resulted in the development of very few female worms, while male worm numbers were unchanged. The few female worms that were able to develop from *Wolbachia*-depleted Mf had endobacteria levels equivalent to control worms indicating that these worms developed from Mf with a *Wolbachia *threshold needed for successful development of the larvae. Thus, the *O. volvulus *L3 that had successfully developed at 4 and 5 months post treatment developed from Mf with the minimum of *Wolbachia *endosymbionts to develop into L3.

In our study the larvae were not exposed to doxycycline within the insect vector. The treatment occurred before the blackflies had fed on the infected volunteers. Because doxycycline rapidly blocks embryogenesis by *Wolbachia *depletion [12], the Mf taken up by the flies during the blood meal could only be those present at the time of doxycycline treatment. Therefore we concluded that *Wolbachia *depletion alone causes the impairment of larval development and is not a direct effect of the antibiotic on the larvae.

As noted, issues of small sample size arose during some of our analyses. Although a clear difference in the number of L3 that developed between the doxycycline and placebo groups could be seen 4 months post-treatment, the difference was not significant (P = 0.088). Because there was a significant difference seen for this parameter at 3 and 5 months, we are confident that with a larger cohort of volunteers the difference seen at 4 months post-treatment would reach significance. Such a larger study should also take into account the drop in L3 development from pre-treatment to five months post-treatment in the placebo group and be planned during a season or an area where the ambient temperature does not differ by 4°C. A larger sample size would also allow us to perform statistics on the quantitative PCR results. We would like to note that the statistics would only strengthen what we have already seen, i.e. larvae which developed from *Wolbachia*-depleted Mf did not have any or had very low levels of *Wolbachia *as detected by quantitative real-time PCR.

## Conclusions

To our knowledge, this is the first study analysing the effects of antibiotic treatment of *O. volvulus *infected humans on larval development in the insect vector. These findings have epidemiological implications. The results clearly show that depletion of *Wolbachia *not only affects the larval stages and adult worms in the mammalian host, it also has negative effects on larval development in blackflies. The decrease in L3 production, even though it is not 100%, could reduce the transmission potential of blackflies following treatment of communities with doxycycline to control onchocerciasis. Thus anti-wolbachial therapy for filarial infections would have an additive effect of interrupting transmission by 1) blocking embryogenesis, 2) macrofilaricidal activity and 3) reduction of the number of L3 that can be transmitted by blackflies that have fed on infected persons before the Mf have been cleared from the skin.

A recent report on community-directed delivery of doxycycline for the treatment of onchocerciasis indicated that the delivery of doxycycline for six weeks is achievable. The therapeutic coverage and the compliance treatment rate achieved in this study coupled to the known efficiency of doxycycline demonstrate that mass administration may be used in selected problem areas such as in areas co-endemic for loiasis to control onchocerciasis [36].

## Competing interests

The authors declare that they have no competing interests.

## Authors' contributions

AA performed DNA extraction and PCR analysis, compiled the data, and drafted the manuscript.

AH developed and monitored the project, analysed data and corrected the manuscript.

KP oversaw PCR assay development, statistical analysis and corrected the manuscript.

MEE participated in patient recruitment, administration of doxycycline and placebo, performed dissection of flies and DNA extraction.

NT participated in patient recruitment, administration of doxycycline and placebo, performed dissection of flies, and performed initial larval recovery statistics.

PE participated in patient recruitment and doxycycline treatment and performed dissection of flies.

SW developed and monitored the project, analysed data and corrected the manuscript.

UK performed statistical analysis.

All authors read and approved the final manuscript.
